# Supplementary material for: Learning curve and surgical outcome of robotic assisted colorectal surgery with ERAS program
Source: Sci Rep. 2022 Nov 29;12:20566. doi: 10.1038/s41598-022-24665-w (PMC9709162; doi:10.1038/s41598-022-24665-w)
Supplement: Supplementary file 1 — Supplementary Information 1. [file 41598_2022_24665_MOESM1_ESM.zip › LSC Raw data-Ñ[▒K/095/O2_ñw╝╨░O▒Kñσ.pdf]

正式摘要(電子簽章：000000200385248)

W55 / W55

臺中榮民總醫院

Operation Note

[redacted]

- 手術日期：2020/09/30
- 手術醫師：VS 吳峰旭
- Anesthesia : GE
- Position : Supine
- Aseptic agents : Soap , Betadine Alcohol

Diagnosis

- Pre-operative diagnosis : GBS, T-colon Ca
- Post-operative diagnosis : Ditto.

Surgical Indication

- for symptoms relief

Operation

- Laparoscopic Cholecystectomy

Finding

- 1. gallbladder, in size of 6x3x3cm, without wall thickenss, no severe adhesion was noted.
  2. One pigmented gallstone noted in Hartmann's punch

Procedure

- GS was table consulted for colecystectomy. The gallbladder was identified and Calot's triangle was dissected. Cystic duct was identified and ligated with clip. Cystic artery was also dissected and resected. The gallbladder was deteched from the liver bed and bleeder was checked. The gallbladder was removed via LLQ wound. CRS took over for finish the operation.
- Estimated blood loss: minimum
- 器械數量: 正確
- 紗布數量: 正確

編輯主治 2215H吳峰旭

正式摘要(電子簽章：000000200385248)

W55 / W55

doctorRank:B
